# Complete genome sequence of *Yersinia pseudotuberculosis* strain SP-1303 from lineage 8, associated with Far East scarlet-like fever

**DOI:** 10.1128/MRA.00838-23

**Published:** 2023-10-31

**Authors:** Marion Lemarignier, Cyril Savin, Pierre Lê-Bury, Olivier Dussurget, Javier Pizarro-Cerdá

**Affiliations:** 1 Institut Pasteur, Université Paris Cité, CNRS UMR6047, Yersinia Research Unit, Paris, Ile de France, France; 2 Institut Pasteur, Université Paris Cité, Yersinia National Reference Laboratory, WHO Collaborating Research & Reference Centre for Plague FRA-140, Paris, Île-de-France, France; Rochester Institute of Technology, Rochester, New York, USA

**Keywords:** *Yersinia pseudotuberculosis*, Far East scarlet-like fever, genome

## Abstract

We report the complete genome sequence of *Yersinia pseudotuberculosis* strain SP-1303, identified as part of lineage 8 and associated with Far East scarlet-like fever. The genome includes the chromosome, the *Yersinia*-virulence plasmid (pYV) encoding a type III secretion system essential for virulence, the pVM82 plasmid, and two cryptic plasmids.

## ANNOUNCEMENT

*Yersinia pseudotuberculosis* is one of the 26 species of the genus *Yersinia*, a member of the *Yersiniaceae* family from the order *Enterobacterales* (1, 2). *Y. pseudotuberculosis* is a foodborne pathogenic bacterium, known to cause self-limiting acute enteropathy in humans, taking the form of mesenteric lymphadenitis and gastroenteritis with fever, abdominal pain, and occasionally diarrhea, which in rare cases takes a septicemic form (3 – 5). The Far East scarlet-like fever (FESLF) is another form of yersiniosis, first reported in 1959 during an epidemic in Vladivostok and mainly present in Far Eastern countries, with symptoms including fever, hyperemic tongue, skin rash, jaundice, and desquamation (6, 7). A single complete genome sequence of *Y. pseudotuberculosis* strain IP31758 causing FESLF was published in 2007 by M. Eppinger and colleagues (8). However, the strain IP31758 does not harbor the *Yersinia*-virulence plasmid (pYV) encoding a type III secretion system essential for virulence, which is often lost under laboratory culture conditions (9), making the IP31758 of limited use. The strain SP-1303 was isolated from mouse stools in 1988 in Moscow. It was recovered by the Shimane Prefectural Institute of Public Health and Environmental Science (Matsue, Japan) and given at −80°C in LB-glycerol to the *Yersinia* Research Unit by Professor H. Fukushima in 2011. The strain SP-1303 belongs to the *Y. pseudotuberculosis* lineage 8 together with strains associated with FESLF (including strain IP31758) and harbors the pYV, allowing for the first time to fully understand the complete repertoire of virulence factors displayed by these strains, which is of major interest to study this poorly characterized disease (10).

Genomic DNA from *Y. pseudotuberculosis* strain SP-1303, grown in LB medium at 28°C for 16 h, was extracted using the PureLink Genomic DNA kit (Invitrogen) according to the manufacturer’s instruction, except the final elution step carried out in H_2_O (Ambion). Two complementary sequencing technologies were applied using the same purified DNA: Oxford Nanopore Libraries (ONT) long reads and Illumina short reads (paired-end reads of 150 nucleotides). Libraries were prepared using the Rapid Barcoding kit (SQK-RBK004) for R9.4.2 ONT MinION flow cell sequencing, and the Nextera kit (Illumina) for Illumina NextSeq 550 sequencing. ONT Guppy v6.4.2 was used for long reads base-calling in high-accuracy mode (configuration dna_r9.4.1_450bps_hac.cfg). All reads were provided as FASTQ files and their information is detailed in Table 1. Hybrid genome assembly with both Illumina and ONT sequencing data was carried out as previously described by R. Wick and colleagues (11). Briefly, reads were filtered with Filtlong (https://github.com/rrwick/Filtlong) (ONT) and fqCleanER (https://gitlab.pasteur.fr/GIPhy/fqCleanER) (Illumina) for assembly using Trycycler/0.5.0 (12), Flye v2.9 (13), Raven/1.6.0 (14), Miniasm/0.3, and Minipolish/0.1.3 (15). Assembled reads were polished with Medaka/1.4.4 (https://github.com/nanoporetech/medaka) (ONT) and Polypolish/0.5.0 (16) (Illumina). Contigs were circularized and rotated by Trycycler/0.5.0 (12). Genome annotation was performed by the NCBI Prokaryotic Genome Annotation Pipeline (PGAP) (17 – 19) (Table 1).

**TABLE 1:** Assembly and general features of *Y. pseudotuberculosis* strain SP-1303

Paramater	Whole genome	Chromosome	pVM82	pYV	pYpstbSP1303.1	pYpstbSP1303.2
GenBank accession no.		CP130901	CP130902	CP130903	CP130904	CP130905
No. of circular contigs	5	1	1	1	1	1
Size (bp)	4,979,983	4,741,829	153,152	74,956	5,546	4,500
G + C content (%)	45.6	47.5	40	44.5	48	48
No. of annotated protein-coding genes	4,186	3,939	161	73	6	7
No. of pseudogenes	155	126	3	24	1	1
No. of rRNAs	22	22	0	0	0	0
No. of tRNAs	84	84	0	0	0	0
No. of ncRNAs	10	10	0	0	0	0
No. of CRISPR arrays	2	2	0	0	0	0
Total no. of reads	17,811 (ONT); 5,355,151 (Illumina)					
ONT reads information (20)	N50 length: 18,828; mean length: 10,193; median length: 6,400; max length: 109,206					
Total no. of bases	181,551,528 (ONT); 1,572,500,161 (Illumina)					
Sequencing depth (x)	36.5 (ONT); 284 (Illumina)					

Our work reveals a circular chromosome of 4.7 Mb encoding the superantigen YpmA (21), the pYV (74.9 kb) plasmid found in pathogenic *Yersinia* species encoding a type III secretion system (22), and the pVM82 (153.1 kb) plasmid also present in the IP31758 FESLF strain, which harbors a type IVB secretion system that could be involved in the pathogenesis of FESLF (8, 23 – 26). Two additional cryptic plasmids were identified with MOB-suite (27, 28): pYpstbSP1303.1 (5.5 kb) and pYpstbSP1303.2 and compared using BLAST. The plasmid pYpstbSP1303.1 is close to plasmids present in *Klebsiella pneumonia* (identity: 98.15%, query cover: 54%), *Escherichia coli* (identity: 97.96%, query cover: 49%), and *Salmonella enterica* (identity: 97.84%, query cover: 49%). The second plasmid pYpstbSP1303.2 (4.5 kb) resembles the pHW66 plasmid of *Rhanella sp.* (identity: 95.24%, query cover: 63%), a ColE-2-like plasmid (29). No antimicrobial resistance genes were detected using ResFinder 4.3.3 (30).

## Data Availability

The genome sequence of *Y. pseudotuberculosis* strain SP-1303 has been submitted to NCBI under the BioProject ID PRJNA998338 and the Biosample accession number SAMN36701287. The assembled genome is available in GenBank under assembly accession number GCA_030644845. Sequencing raw reads are available on Sequencing Read Archive (SRA) accession numbers SRR25411577 (Illumina) and SRR25411576 (ONT). In addition, the genome sequence is also available on Yersiniomics, a multi-omics interactive web-based platform for easy visualization of genome organization, rapid access to structural and functional protein properties, and search for protein homologs within the *Yersinia* genus (31).
